# Cytoplasmic Acidification and the Benzoate Transcriptome in *Bacillus subtilis*

**DOI:** 10.1371/journal.pone.0008255

**Published:** 2009-12-14

**Authors:** Ryan D. Kitko, Rebecca L. Cleeton, Erin I. Armentrout, Grace E. Lee, Ken Noguchi, Melanie B. Berkmen, Brian D. Jones, Joan L. Slonczewski

**Affiliations:** 1 Department of Biology, Kenyon College, Gambier, Ohio, United States of America; 2 Department of Chemistry and Biochemistry, Suffolk University, Boston, Massachusetts, United States of America; 3 Department of Mathematics, Kenyon College, Gambier, Ohio, United States of America; University of Liverpool, United Kingdom

## Abstract

**Background:**

*Bacillus subtilis* encounters a wide range of environmental pH. The bacteria maintain cytoplasmic pH within a narrow range. Response to acid stress is a poorly understood function of external pH and of permeant acids that conduct protons into the cytoplasm.

**Methods and Principal Findings:**

Cytoplasmic acidification and the benzoate transcriptome were observed in *Bacillus subtilis*. Cytoplasmic pH was measured with 4-s time resolution using GFPmut3b fluorimetry. Rapid external acidification (pH 7.5 to 6.0) acidified the *B. subtilis* cytoplasm, followed by partial recovery. Benzoate addition up to 60 mM at external pH 7 depressed cytoplasmic pH but left a transmembrane ΔpH permitting growth; this robust adaptation to benzoate exceeds that seen in *E. coli*. Cytoplasmic pH was depressed by 0.3 units during growth with 30 mM benzoate. The transcriptome of benzoate-adapted cells was determined by comparing 4,095 gene expression indices following growth at pH 7, +/− 30 mM benzoate. 164 ORFs showed ≥2-fold up-regulation by benzoate (30 mM benzoate/0 mM), and 102 ORFs showed ≥2-fold down-regulation. 42% of benzoate-dependent genes are regulated up or down, respectively, at pH 6 versus pH 7; they are candidates for cytoplasmic pH response. Acid-stress genes up-regulated by benzoate included drug resistance genes (*yhbI*, *yhcA*, *yuxJ*, *ywoGH*); an oligopeptide transporter (*opp*); glycine catabolism (*gcvPA*-*PB*); acetate degradation (*acsA*); dehydrogenases (*ald*, *fdhD*, *serA*, *yrhEFG*, *yjgCD*); the TCA cycle (*citZ*, *icd*, *mdh*, *sucD*); and oxidative stress (OYE-family *yqjM*, *ohrB*). Base-stress genes down-regulated by benzoate included malate metabolism (*maeN*), sporulation control (*spo0M*, *spo0E*), and the SigW alkali shock regulon. Cytoplasmic pH could mediate alkali-shock induction of SigW.

**Conclusions:**

*B. subtilis* maintains partial pH homeostasis during growth, and withstands high concentrations of permeant acid stress, higher than for gram-negative neutralophile *E. coli*. The benzoate adaptation transcriptome substantially overlaps that of external acid, contributing to a cytoplasmic pH transcriptome.

## Introduction

*Bacillus subtilis* encounters a wide range of extracellular pH values during growth in various environments, including diverse soils, association with plants, and within the gastrointestinal tract [1], [2]. The bacteria maintain cytoplasmic pH within a narrow range that allows for the stability of nucleic acids and proteins and subsequent growth over several log units of environmental pH [3]–[5]. *Bacillus* species respond to environmental pH in ways that are important for pathogenesis. For example, food-borne *B. cereus* encounters acidic environments in the gastrointestinal tract and in food products [6]–[9]. Low external pH magnifies the inhibitory effects of membrane-permeant organic acids such as acetic, benzoic and salicylic acids; but the relationship between pH and the organic compound is often unclear in the literature [9]–[11]. A permeant acid stresses the cell by importing protons, depressing cytoplasmic pH, and by concentrating the organic anion within the cytoplasm in proportion to the transmembrane pH difference (ΔpH) [12]–[16]. Thus, the effects of external pH, cytoplasmic pH, and any exogenous or endogenous permeant acids must be considered together.

Permeant acids are ubiquitous in the environment of heterotrophic bacteria. Many permeant acids are products of fermentation, such as acetic, formic and propionic acids. Others are phenolic acids in soil, such as plant metabolites and degradation products of polycyclic aromatic hydrocarbons (PAH) [17]. Benzoic, salicylic, and sorbic acids are commonly used by the food industry as preservatives to inhibit food spoilage. Permeant acids that depress cytoplamic pH and concentrate within cells at low external pH decrease bacterial proton potential, activate stress responses, and up-regulate drug resistance complexes [14], [15], [18]–[21]. Sorbate stress up-regulates urease (*ureABC*), *padC*, and the tricarboxylic acid (TCA) cycle producing a transcriptomic response similar to that of nutrient limitation [11]. Salicylate up-regulates urease, aminomethyltransferase (*gcvT*), and both phenolic acid decarboxylases (*padC* and *bsdBCD*) and down-regulates ATPases [10]. *Bacillus* species can metabolize some permeant acids, for example ferulic, *p*-coumaric, and caffeic acids via the phenolic acid decarboxylases such as PadC [22], [23].

Nevertheless, the role of pH in permeant-acid effects on *Bacillus* species remains poorly understood. Often studies of such acids are conducted under conditions in which pH stress is not clearly distinguished from the effects of the organic molecule. For example, Van Duy *et al*. (2007) added salicylic acid to unbuffered cultures causing a drop of two units in external pH (from pH 7 to 5.5) [10]; thus, the reported salicylate transcriptome includes external acid shock.

Cytoplasmic pH homeostasis in *Bacillus* species has been studied extensively in the neutral to alkaline range [4], [24]. Krulwich, Padan and colleagues document the role of sodium and proton transport through transporters such as Mrp and TetL in maintaining cytoplasmic pH at high external pH, where the ΔpH is inverted [4], [24]–[26]. Alkali shock induces the SigW regulon by a mechanism involving proteolysis of the membrane-embedded anti-sigma factor RsiW [27]–[31]. By contrast, relatively little is known about *B. subtilis* pH homeostasis in the acidic range [3]. A transcriptomic study compares log-phase cultures buffered at pH 6, pH 7, and pH 9, showing that acid up-regulates acetoin production, catabolic dehydrogenases and decarboxylases, and the SigX regulon [31]. During sporulation, pH of the forespore falls by a full unit [32], followed by pH rise during germination [33]. Thus, pH in the acid range raises important questions for *Bacillus* development.

Here we report on the effects of external acid stress and benzoate stress on cytoplasmic pH. We developed a fluorimetry vector based on GFPmut3b [34] to measure *B. subtilis* cytoplasmic pH with 4-s time resolution. Our method, based on a similar approach in *E. coli* [35], has substantial advantages over previous measurements of *B. subtilis* pH using the equilibration of radiolabeled permeant acids and bases [3], [32], [33] and fluorescent dyes that require loading and microscopy [36], [37]. We followed up our measurements with a transcriptomic study of benzoate adaptation at external pH 7, conducted under conditions enabling direct comparison with the external-acid adaptation transcriptome [31]. Both conditions include comparable exposure to fermentation acids generated from the growth medium, but the benzoate provides substantially greater permeant acid concentration. Both conditions incur a significant decrease of cytoplasmic pH, but the benzoate experiment maintains external pH 7. Thus, the overlap between benzoate and external-acid transcriptomes may represent a “cytoplasmic pH” transcriptome.

## Results

### GFPmut3b Serves As a Reliable Reporter for Cytoplasmic pH of *B. subtilis* Cell Suspensions

**Figure 1:**
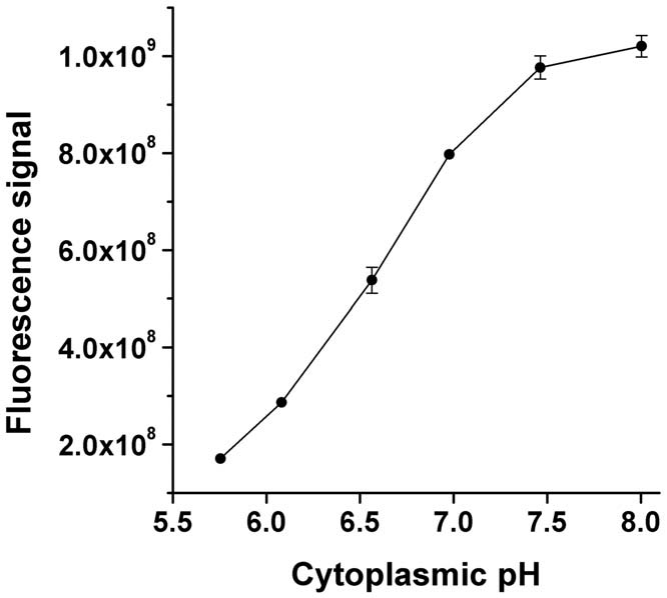
Fluorescence signal as a function of cytoplasmic pH. *B. subtilis* MMB1311 was cultured in buffered LBK to OD_600_ 0.2 and resuspended at OD_600_ 0.4 in supplemented M63 medium buffered as described in the Materials and Methods, with inclusion of 10 µM nigericin. Fluorescence intensity was summed over the excitation range of 480–510 nm, with emission at 545 nm. The mean intensity for three independently grown cultures is shown, with error bars representing SEM.

To measure cytoplasmic pH of *B. subtilis*, a reporter plasmid was constructed with a promoter appropriate for this organism (P*bsr*) and a gene encoding a high-intensity fluorophore GFPmut3b. The pH dependence of fluorescence excitation spectra of GFPmut3b was determined by observation of cultures adjusted to various pH values in the presence of nigericin, a K^+^/H^+^ antiporter that equalizes cytoplasmic and extracellular pH [38]. Log-phase cultures of *B. subtilis* MMB1311 were resuspended at OD_600_ 0.4 in buffered supplemented M63 medium adjusted to pH values over the range of pH 5.5 to pH 8.0. 10 µM nigericin was added prior to fluorimetry. Trials with higher concentrations (up to 100 µM) at pH 6.0 indicated no further collapse of ΔpH (data not shown). The fluorescence signal increased with pH in a near-linear model over the range of pH 6–7, with a slope decreasing from pH 7–8 (Fig. 1). This pH dependence is consistent with previous observations of GFPmut3 *in vitro* [34] or expressed within *E. coli* [35]. We conclude that GFPmut3 serves as a reliable reporter of cytoplasmic pH in *B. subtilis* in the pH range of 5.5–8.0.

### Addition of HCl Shifts Cytoplasmic pH

**Figure 2:**
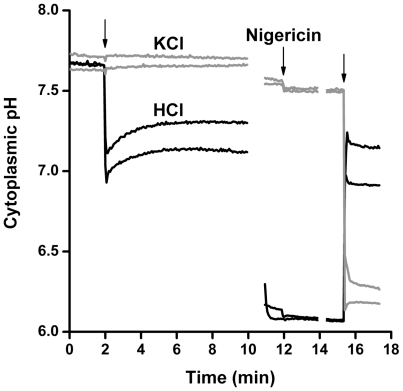
Effect of external acid shift on cytoplasmic pH. MMB1311 was cultured in buffered LBK, then resuspended at OD_600_ 0.4 in M63 supplemented with casein hydrolysate (5 mM MES, 5 mM MOPS, pH 7.5). At 2 min, 15.5 mM HCl was added to shift the external pH from pH 7.5 to pH 6.0. (15.5 mM KCl was used as a control for volume and osmotic effects). At 11–12 min, 10 µM nigericin was added to fully collapse ΔpH for the individual replicate standard curve calculations. To obtain a second standard curve point, 12.5 mM KOH was added to raise the pH for the HCl curves and 15.5 mM HCl was added to lower the pH for the KCl curves. Each condition is represented by two out of three independent cultures. Gaps in the data sets represent the time it took to set up for the next phase of the experiment. Fluorescence intensity was converted to pH units using the internal standard curves as described in Materials and Methods.

*B. subtilis* maintains steady-state pH homeostasis, with a modest degree of external pH dependence, over the range of external pH 6–8 [3]. The response of cytoplasmic pH to rapid acidification has been reported in *E. coli* [35], [39] but not in *B. subtilis*. We observed the perturbation and recovery of cytoplasmic pH following rapid acidification of the external medium (Fig. 2). *B. subtilis* MMB1311 was suspended in buffered M63 medium (5 mM 3-(*N*-morpholino)propanesulfonic acid [MOPS], 5 mM 2-(*N*-morpholino)ethanesulfonic acid [MES]) at pH 7.5. After equilibration for 2 min, 15.5 mM HCl was added to lower the external pH to approximately pH 6.0, which was confirmed by direct pH meter measurement. For control cultures, the same concentration of KCl was added. KCl addition had no significant effect on fluorescence intensity.

**Figure 3:**
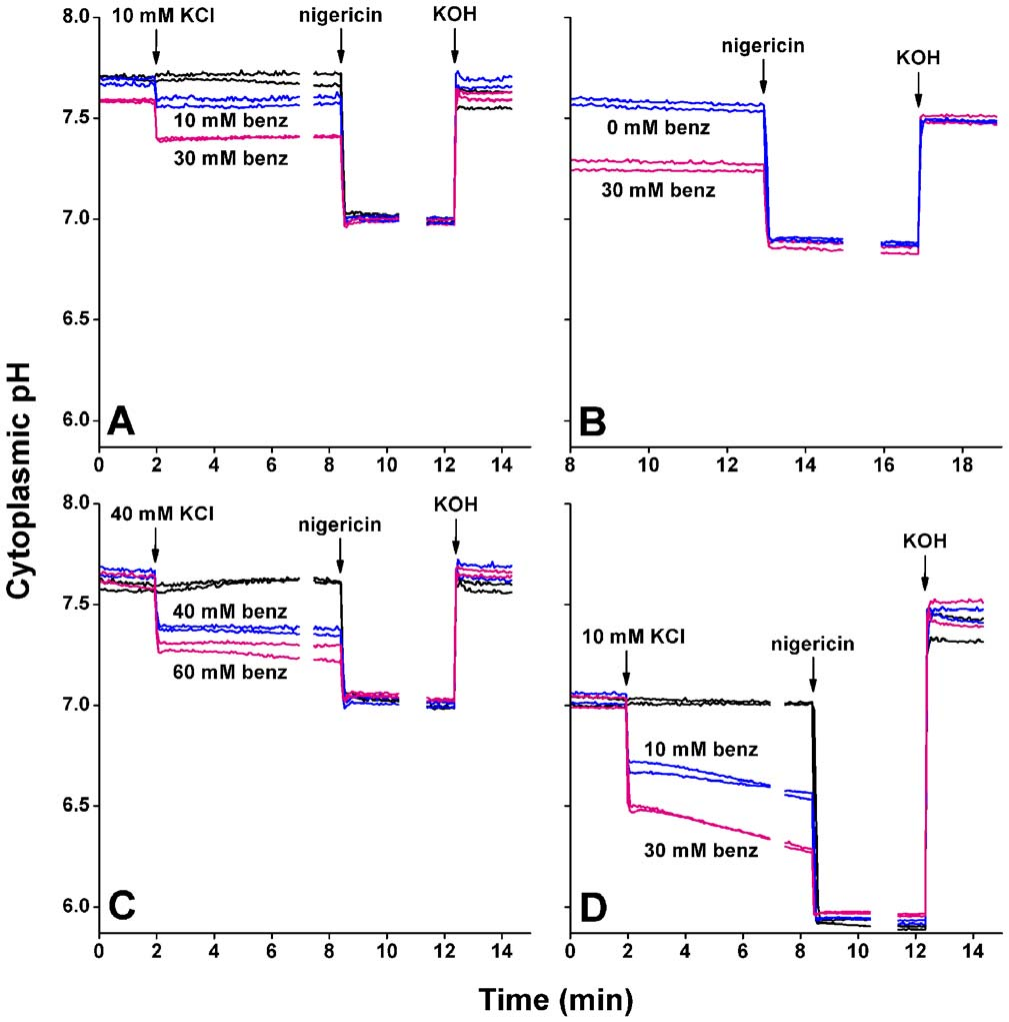
Effect of various concentrations of sodium benzoate on cytoplasmic pH. MMB1311 was cultured in buffered LBK, then resuspended at OD_600_ 0.4 in buffered M63 medium with casein hydrolysate. (**A**) Grown at pH 7.0 (50 mM MOPS); resuspended at pH 7.0 (5 mM MOPS). (**B**) Grown at pH 7.0 (50 mM MOPS) with or without 30 mM sodium benzoate; resuspended at pH 6.9 (5 mM MOPS) with or without sodium benzoate. (**C**) Grown at pH 7.0 (50 mM MOPS); resuspended at pH 7.0 (5 mM MOPS). (**D**) Grown at pH 6.0 (50 mM MES); resuspended at pH 6.0 (5 mM MES, 5 mM MOPS). For all panels, 10 µM nigericin was then added to fully collapse ΔpH for the individual replicate standard curve calculations. To obtain a second standard curve point, KOH (5.5 mM for **A**, **B**, and **C**, and 12.5 mM for **D**) was added to raise the pH. Each condition is represented by two out of three independent cultures.

Prior to external acid treatment, the cytoplasmic pH of acid-treated cultures was pH 7.7 (Fig. 2). This pH value was determined by interpolating between two pH values measured with nigericin at the end of the experiment. After HCl addition, the cytoplasmic pH of each sample fell within 8 s to pH 6.9–7.1. Cytoplasmic pH recovery began immediately after the lowest recorded pH and continued steadily for approximately 3.5 min, ultimately recovering to pH 7.1–7.3. This value of recovered cytoplasmic pH was comparable to that seen in cells grown and resuspended in medium buffered at pH 6 (Fig. 3D).

### Cytoplasmic pH Decreases in the Presence of Benzoate

Organic weak acids such as benzoate derivatives depress cytoplasmic pH during steady-state growth [13], [14], [35]. In *E. coli*, benzoate addition partly or fully depresses internal pH; if partial ΔpH remains, then benzoate is taken up in proportion to the remaining ΔpH [14]. Similar pH effects are expected in *B. subtilis* although they have not been observed directly. We observed the effect of rapid addition of various concentrations of sodium benzoate on cytoplasmic pH (Fig. 3). *B. subtilis* MMB1311 cultures were suspended in M63 medium buffered at either pH 7.0 (Fig. 3A) or pH 6.0 (Fig. 3C). After two minutes of initial measurements, sodium benzoate was added to a final concentration, with no change in extracellular pH. Similar concentrations and volumes of KCl were used as controls for osmotic or volume addition effects; no significant change in fluorescence signal was observed.

At external pH 7.0, *B. subtilis* maintained a cytoplasmic pH between 7.6 and 7.7 (Fig. 3A, 3B, and 3C). Addition of 10 mM benzoate lowered the cytoplasmic pH by approximately 0.1 unit, whereas 30 mM benzoate decreased the cytoplasmic pH by 0.3 units (Fig. 3A). Similar values of cytoplasmic pH were observed for cultures grown in buffered LBK pH 7 with or without 30 mM benzoate, before resuspension in M63 supplemented medium (Fig. 3B). The cells grown with 30 mM benzoate also showed cytoplasmic pH values 0.3 units lower than those of the control culture.

Higher levels of benzoate concentration were also tested. At external pH 7, the addition of 40 mM benzoate resulted in the reduction of the cytoplasmic pH by 0.3 pH units, whereas 60 mM benzoate lowered it by 0.4 units (Fig. 3C). At 60 mM benzoate, a residual value of ΔpH (0.25 units) remained.

At lower external pH, the permeant acid would be expected to depress cytoplasmic pH to a greater extent. Cells suspended at pH 6.0 showed cytoplasmic pH at pH 7.0–7.1 (Fig. 3D). This value was comparable to the value reached after a sudden shift in external acid from pH 7.5 to pH 6.0 (Fig. 2). At external pH 6, the initial cytoplasmic pH was significantly lower than the cytoplasmic pH maintained at external pH 7 (see Fig. 3A), but the cells still maintained substantial pH homeostasis, with a full unit of ΔpH (Fig. 3D). Addition of 10 mM or 30 mM benzoate lowered the cytoplasmic pH to pH 6.5–6.7. In this case, cytoplasmic pH continued to fall for several minutes after the initial shift. For comparison, in *E. coli* [35], comparable addition of benzoate at pH 6 depresses the cytoplasmic pH rapidly and completely. Thus, *B. subtilis* showed a stronger ability to maintain cytoplasmic pH than does the gram-negative neutralophile.

A mechanism of cytoplasmic pH homeostasis under acid stress may involve K^+^ or Na^+^ transport, although the evidence remains unclear [5]. We tested whether pH response to benzoate stress requires high external K^+^ or Na^+^ by repeating the experiment of Fig. 3C (40 mM benzoate addition) using cells suspended in supplemented M63 medium with K^+^ and Na^+^ concentration limited to 10 mM. Both sodium benzoate and potassium benzoate added to 40 mM gave essentially the same results as seen in Fig. 3C; the equivalent cytoplasmic acidification profile was seen for benzoate addition in the presence of 100 mM KCl (data not shown). Thus, while a role for ion transport cannot be ruled out, no more than 10 mM K^+^ or Na^+^ was required for partial cytoplasmic pH maintenance in the presence of 40 mM benzoate.

### Benzoate Inhibits *B. subtilis* Growth

To prepare for transcriptomic experiments, the growth rate of benzoate-adapted cells was tested. An overnight culture of *B. subtilis* AG174 was diluted 500-fold into LBK buffered with 50 mM MOPS at pH 7.0 containing 0, 10, 30, or 60 mM sodium benzoate. For each condition, three independent cultures were observed. Maximal doubling times during logarithmic growth were as follows: 19±1 min for 0 mM benzoate, 22±0.2 min for 10 mM benzoate, 28±0.8 min for 30 mM benzoate, and 50±2 min for 60 mM benzoate. Thus, concentrations between 10–60 mM significantly stressed the cells; yet log-phase growth occurred at a benzoate concentration as high as 60 mM. If the ΔpH is maintained at 0.25 units (as measured above, Fig. 3D), then cells growing with 60 mM benzoate contain about 100 mM intracellular benzoate. By contrast, in *E. coli* at pH 7, 40 mM benzoate rapidly eliminates ΔpH with no recovery, and no growth [35].

### The Benzoate Transcriptome Overlaps the External-Acid Transcriptome

Our results showed that benzoate addition at external pH 7 acidifies the cytoplasm by several tenths of a unit, a change comparable to that observed following external acid shift, either rapid or steady-state [3]. We hypothesized that the set of genes responsive to benzoate would overlap the set of genes responsive to external pH in the mild acid range (pH 6 versus pH 7) as reported [31]. The genes responsive both to benzoate and to low external pH would be likely candidates for cytoplasmic pH response and involvement in cytoplasmic pH homeostasis. No previous study of permeant-acid stress effectively isolates and compares the effects of a permeant acid versus external pH.

To test the above hypothesis, we measured transcriptomic gene expression ratios during steady-state growth with or without benzoate. Steady-state conditions were chosen, in preference to a time course following benzoate addition, for the following reasons. The steady-state design enabled direct comparison between benzoate adaptation and the adaptation to low external pH as reported recently [31]. Furthermore, in our experience, a rapid-stress transcriptome typically includes a large proportion of universal stress genes that yield little information about the specific stress condition tested. For example, Van Duy *et al*. (2007) showed that salicylate mainly induces the CtsR and SigB general stress regulons [10].

For the benzoate transcriptome, four replicate cultures were grown to early log phase in the presence or absence of 30 mM benzoate, as described under Methods. Prior to the RNA extraction, the doubling times observed at pH 7.0 were 19±1 min for 0 mM benzoate cultures, and 33±2 min for 30 mM benzoate cultures. The cDNA from four independent cultures of the 0 mM and 30 mM sodium benzoate conditions was hybridized to Affymetrix antisense *B. subtilis* arrays. Array data were deposited at the NCBI Gene Expression Omnibus (GEO accession no. GSE12263). To test for significant differences in expression between the experimental conditions, we conducted a two-sample t-test on the log_2_-transformed model-based expression indices on a gene-by-gene basis. The t-tests were performed at a significance level of 0.001, meaning approximately one false positive would be expected per 1000 genes tested. Expression ratios were determined and presented here in the form of 30 mM benzoate/0 mM benzoate, so that the log_2_ ratios are positive for genes expressed more in the presence of benzoate and negative for genes expressed less. Values presented are significant based on the two-sample t-test (*P*≤0.001) (see Table S1A).

Of the approximately 4,095 unique protein-encoding ORFs in *B. subtilis* included on the Affymetrix chip, 164 genes showed increased expression ratios of two-fold or greater (log_2_ ratio, ≥1) in the presence of benzoate, whereas 102 genes showed decreased expression ratios of twofold or greater. Overall, 477 genes (corresponding to 499 probe sets), or 11.6% of the genome, showed significant expression differences in the presence of 30 mM benzoate (Table S1A). Intergenic regions showing significant expression in the presence of benzoate (28 intergenic probe sets out of 606 on the array) are presented in Table S1B.

The genes up- or down-regulated in the presence of benzoate could respond to the effect of benzoate anion accumulation within *B. subtilis* (such as its interaction with the membrane or the accumulation of toxic anions in the cytoplasm) or to the depression of cytoplasmic pH and subsequent partial collapse of the transmembrane ΔpH, or to both. Of all the genes we found to be up-regulated by benzoate, 42% show up-regulation during growth at external pH 6 compared to pH 7 [31]. Of genes down-regulated by benzoate, 43% show down-regulation at pH 6.

**Figure 4:**
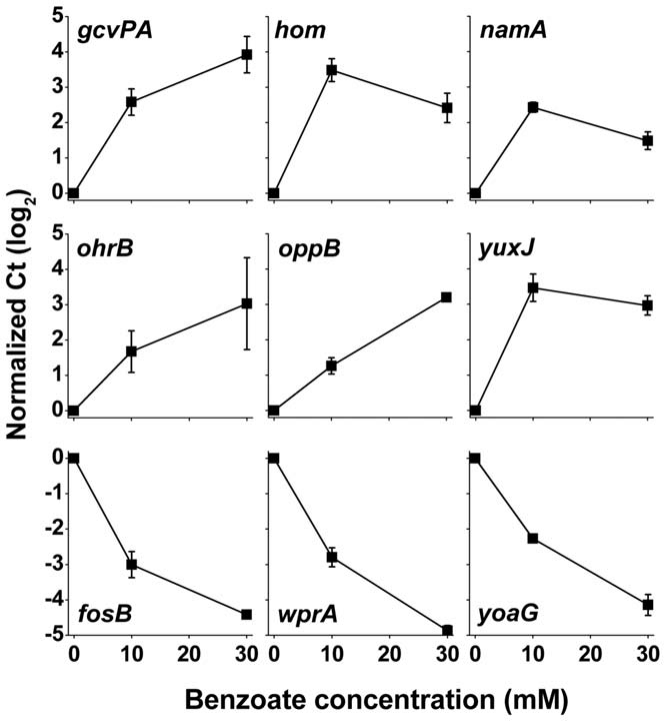
Real-time PCR expression ratios of selected genes as a function of benzoate concentration. *B. subtilis* strain AG174 overnight cultures in unbuffered LBK were diluted 500-fold into LBK buffered with 50 mM MOPS with 0, 10, or 30 mM sodium benzoate. RNA was isolated and mRNA expression for individual genes was quantified by real-time PCR using an ABI Prism 7500 DNA analyzer (Applied Biosystems) with SYBR Green one-step protocol. All expression levels are presented relative to the expression at 0 mM benzoate. Error bars represent the SEM (*n* = 3).

Table 1 ranks the most highly responsive genes, those that were up-regulated or down-regulated at least fourfold in the presence of benzoate (log_2_ ratio ≥2). The regulated genes are organized as operons and regulons in Table 2. For nine genes of interest, expression ratios were confirmed using real-time PCR (Fig. 4). All genes tested by PCR showed expression ratios consistent with those observed in the arrays.

**Table 1:** Genes up-regulated or down-regulated fourfold or greater in the presence of 30 mM benzoate.

		Log_2_ expression ratios		Regulon^c^	
Gene^a^	Description	30/0 mM	pH 6/7^b^	+	-
**Up-regulated**					
*padC*	Phenolic acid decarboxylase	4.8			PadR
*yveG*	Unknown	4.7			
*yveF*	Unknown	4.3			
*oppB*	Oligopeptide ABC transporter	3.8	1.6	TnrA	Hpr
*ilvH*	Acetolactate synthase	3.8	1.3	CcpA, TrnS-Leu2	TnrA
*oppD*	Oligopeptide ABC transporter	3.4	1.5	TnrA	Hpr
*ywoH*	MarR family transcriptional regulator	3.3	0.4		
*oppC*	Oligopeptide ABC transporter	3.3	1.2	TnrA	Hpr
*oppA*	Oligopeptide ABC transporter	3.2	1.8	TnrA	Hpr
*oppF*	Oligopeptide ABC transporter	3.2	1.5	TnrA	Hpr
*gcvPA*	Probable glycine decarboxylase	3.2	1.1		
*ywoG*	Antibiotic resistance protein homolog	3.1	0.5		
*yrhG*	Formate transporter homolog	3.1			
*cimH*	Citrate/malate transporter	3.0			CcpA
*yclM*	Probable aspartokinase	2.9			
*ilvC*	Ketol-acid reductoisomerase	2.9	0.8	CcpA, TrnS-Leu2	TnrA
*ylbP*	N-acetyltransferase homolog	2.9			
*yfmO*	Multidrug efflux transporter	2.9			
*ilvB*	Acetolactate synthase	2.8	0.6	CcpA, TrnS-Leu2	TnrA
*gcvT*	Aminomethyltransferase	2.8	0.5		
*gcvPB*	Probable glycine decarboxylase	2.8	0.5		
*ybbH*	HTH-type transcriptional regulator	2.7			
*ybeC*	Amino acid transporter homolog	2.7	1.0		
*citZ*	Citrate synthase II	2.6	0.4		CcpA, CcpC
*ilvD*	Dihydroxy-acid dehydratase	2.6			CodY
*yuxJ*	Multidrug-efflux transporter	2.6	1.1		
*yusY*	Oligoendopeptidase F homolog	2.4	0.3		
*mpr*	Extracellular metalloprotease	2.4			
*yrkN*	Unknown	2.4			
*yfmB*	Unknown	2.3			
*yhcB*	Trp repressor binding protein	2.3	1.3	ComK	
*yrkO*	Unknown	2.3			
*yhaA*	Aminoacylase homolog	2.3	0.6		
*ybbF*	Sucrose phosphotransferase	2.3			
*yfmP*	HTH-type transcriptional regulator	2.3			
*purS*	Purine biosynthesis	2.2			PurR
*acsA*	Acetyl-coenzyme A synthetase	2.2			CcpA, CodY
*purF*	Amidophosphoribosyltransferase	2.2			PurR
*ydjL*	L-iditol 2-dehydrogenase homolog	2.2	0.9	SigB	
*citB*	Aconitate hydratase	2.2		AbrB, SigA, TnrA	CcpC, CodY
*yhbJ*	Unknown	2.2	1.5	ComK	
*yhbI*	MarR family transcriptional regulator	2.2	1.5	ComK	
*yraN*	HTH-type transcriptional regulator	2.1			
*ilvA*	Threonine dehydratase	2.1			CodY
*yhcC*	Unknown	2.1	1.2	ComK	
*ald*	Alanine dehydrogenase	2.1	1.1		
*yusX*	Oligoendopeptidase homolog	2.1	1.0		
*leuA*	2-isopropylmalate synthase	2.1		CcpA, TrnS-Leu2	TnrA
*purQ*	Purine biosynthesis	2.1			PurR
*purC*	Purine biosynthesis	2.0			PurR
*ycsF*	Lactam utilization protein homolog	2.0		SigK, TnrA	KipR
*yhcA*	Multidrug resistance protein	2.0	1.5	ComK	
*yxaC*	Unknown	2.0		SigB	
*yrhP*	Efflux protein homolog	2.0			
*yxeK*	Putative monooxygenase	2.0			YjbD, YrzC
**Down-regulated**					
*yoaG*	Unknown	−3.3	−1.9	SigW	
*fosB*	Metallothiol transferase	−2.7	−1.8	SigW	
*yjoB*	Cell division cycle CDC48	−2.6	−1.9	SigW	
*yxaI*	Unknown	−2.5			
*yxiG*	Unknown	−2.4	−1.3		
*yxiF*	Unknown	−2.4	−1.6		
*ydjQ*	Peroxidase	−2.4	−1.8	SigW	
*wprA*	Cell wall-associated protease	−2.3	−2.0	YvrH	
*yxzG*	Unknown	−2.3	−1.5		
*yknZ*	ABC transporter permease	−2.3	−1.7	SigW	AbrB
*yxzC*	Unknown	−2.2	−1.3		
*srfAA*	Surfactin synthetase	−2.2	−2.5	ComA, PerR	CodY
*pspA*	Phage shock protein A homolog	−2.2	−2.2	SigW	
*maeN*	Na^+^/malate symporter	−2.2	−2.6	YufM	
*yxiI*	Unknown	−2.2	−1.5		
*yknY*	ABC transporter homolog	−2.1	−1.9	SigW	AbrB
*yknX*	Unknown	−2.1	−1.9	SigW	AbrB
*yjcM*	Unknown	−2.1	−2.1		
*yxxG*	Unknown	−2.1	−0.9	YvrH	DegU
*yuxG*	Sorbitol-6-phosphate 2-dehydrogenase	−2.0	−1.6		
*spo0M*	Sporulation-control gene	−2.0	−1.8	SigW, SigH	
*yueB*	Bacteriophage SPP1 adsorption protein	−2.0			
*yfiE*	Unknown	−2.0			

a Significant log_2_ expression ratio values are shown (p≤0.001). For genes represented by duplicate probes in the array, only the promoter-proximal probe is presented.

b Expression ratios at pH 6 versus pH 7 [31] are indicated as +, higher at pH 6; –, higher at pH 7.

c Genes within known sigma factor regulons [28], [68], [69] are indicated: SigB, σ^B^; SigW, σ^W^; SigH, σ^H^. All other positive and negative regulation was obtained from the DBTBS database (http://dbtbs.hgc.jp/).

**Table 2:** Operon expression regulated by 30 mM benzoate.

Regulon^a^	Operon or group^b^	Function	pH 6/7^c^	
**Up-regulated**				
BkdR, SigL	*ptb-bcd-buk-lpdV-bkdAABB*	Branched-chain fatty acid synthesis		
CcpA	*ilvBHC-leuAB*	Branched-chain amino acid synthesis		+
CcpA, CcpC	*citZ-icd-mdh*, *sucCD*	TCA cycle		+
PadR	*yveFG-padC*	Phenolic acid degradation		
PurR	*purEKBCSQLFMNHD*	Purine biosynthesis		
SigB	*yfkJIH*	Stress resistance		+
TnrA	*oppABCDF*	Oligopeptide transport		+
TnrA, CodY	*ureABC*	Urease		
Unknown	*gcvT-gcvPA-gcvPB*	Glycine catabolism		+
	*yfkABC*	Putative mechanosensitive channel		+
	*czcDO*	Potassium and divalent cation transport		+
	*citST-yflP*	Mg^2+^-citrate two-component regulator		
	*fdhD, yjgCD, yrhEFG*	Formate dehydrogenases		+
**Down-regulated**				
SigW, AbrB	*yknWXYZ*	Putative ABC transporter		-
	*sigW-rsiW*	RNA polymerase ECF-type sigma		-
SigW	*yqeZ-yqfAB*	Antimicrobial compound resistance		-
	*ydbST*	Antimicrobial compound resistance		-
YvrH, DegU	*wapA-yxxG*	Cell wall-associated protein		-
Unknown	*liaIH*	Responds to cell wall-active antibiotics		-

a Operon or gene group regulation was obtained from the DBTBS database (http://dbtbs.hgc.jp/).

b Genes showing significant expression ratios (p≤0.001). Some operons are tentative based on bioinformatic assignment. Others are listed by group according to functional similarity.

c Expression ratios at pH 6 versus pH 7 [31] are indicated as +, higher at pH 6; –, higher at pH 7.

### Cytoplasmic Acidification Plays a Role in the Benzoate Stress Response

To distinguish the roles of cytoplasmic pH versus cytoplasmic benzoate accumulation in mediating benzoate stress, we compared our results with a previous study of external acid stress, which also includes cytoplasmic acidification [31]. In our previous study, the *B. subtilis* transcriptome was compared during steady state growth in pH 6.0 medium versus pH 7.0. The current benzoate adaptation transcriptome showed significant regulation for many of the same genes (122 significantly up-regulated and 75 significantly down-regulated) when compared to the pH 6.0/pH 7.0 transcriptome (Table S1A). Genes regulated both by benzoate and by external-acid are good candidates for mediation by cytoplasmic pH.

**Figure 5:**
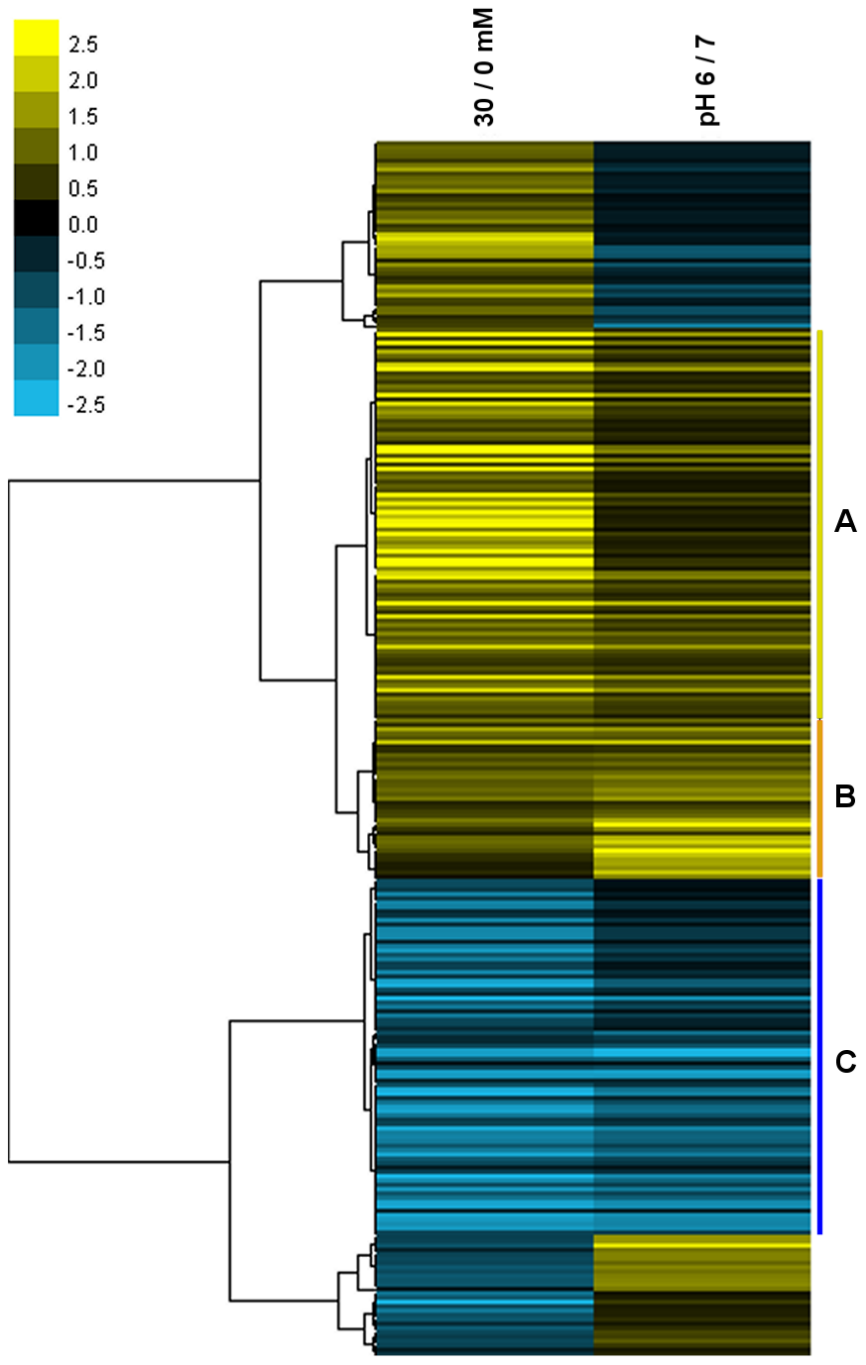
Cluster analysis of gene expression from the benzoate and acid transcriptomes. Hierarchical clustering of probe sets found to be significantly regulated in both the benzoate and previous low pH transcriptomes. Bars in yellow and blue indicate genes that are up- or down-regulated, respectively. Color intensities are proportional to the variation of expression ranging from −2.5 to 2.5 as indicated by the color bar. The group of genes labeled A (vertical yellow bar) indicates those genes up-regulated more by benzoate than by acid and the group labeled B (orange bar) indicates those genes that are up-regulated more by acid than benzoate. The cluster labeled C (blue bar) includes genes that were down-regulated by both benzoate and external acid. A full list of genes included in this analysis can be found in Table S1C.

The comparison of benzoate and external-acid response genes was analyzed further by hierarchical cluster analysis and TreeView visualization (Fig. 5; see Table S1C for full gene annotation). The cluster analysis was used to compare genes with significant expression ratios in both the benzoate and external acid microarrays (280 probe sets in all). The comparison revealed two types of clusters: those that share a parallel response (209 probe sets, 75%) and those that exhibit an opposite response (71 probe sets, 25%) in the two data sets. The largest cluster was that showing up-regulation in response to both benzoate and low pH (127 probe sets, 46%). The cluster up-regulated by both conditions included a subset of 90 probe sets (group A) up-regulated more by benzoate than by external acid, and a smaller subset of 37 probe sets (group B) up-regulated more by external acid than by benzoate, with respect to each experiment's control culture at pH 7. Another cluster showed down-regulation in both conditions (group C, 82 probe sets, 29%).

## Discussion

We present the first dynamic measurements of *B. subtilis* cytoplasmic pH on a rapid time scale, under conditions of acid stress and benzoate stress. Bacteria were cultured in a broth medium (LBK) which, based on our long experience, provides a large range of potential metabolic options for reversing pH stress [5]. The resuspension medium for fluorimentry (supplemented M63) differs somewhat in composition from the growth medium, but includes multiple amino acids available as in LBK. Our results for rapid acid treatment and for steady-state growth in LBK with acid stress were generally consistent, in that the cytoplasmic pH levels obtained following extended growth in buffered LBK were similar to those following a rapid external pH shift after resuspension in supplemented M63 (Figs. 2, 3).

The cytoplasmic pH of *B. subtilis* showed substantial homeostasis, but also a significant difference between cultures grown and resuspended at pH 6 (Fig. 3D) as compared to pH 7 (Fig. 3A). In *B. subtilis*, the cytoplasmic pH showed less recovery from rapid acid shift than that of *E. coli*, which displays a rapid and biphasic recovery [35]. Nevertheless, at either pH 6 or pH 7, *B. subtilis* pH homeostasis withstood a greater degree of benzoate stress than *E. coli.* Although *B. subtilis* grows poorly below external pH 6, its ability to grow and maintain a residual pH under benzoate stress (at or above external pH 6) exceeds that of *E. coli*. The reasons for the difference in benzoate resistance remain to be determined. *Bacillus subtilis* does have the capacity to metabolize benzoate, but it is unlikely that the added benzoate was significantly degraded during the short time scale of our experiments, in which nonaromatic carbon sources were available. The slightly larger cell size of *B. subtilis* compared to *E. coli* results in a lower surface-to-volume ratio, which could decrease the rate of proton accumulation in the cytoplasm, explaining the lack of the “overshoot” of cytoplasmic pH under rapid acid treatment as well as the ability to withstand high benzoate concentrations. *Bacillus subtilis* could also have more active efflux pumps for benzoate. Yet another possibility is that *B. subtilis* trades a slight decrease in cytoplasmic pH (as seen in Fig. 3D) for greater exclusion of benzoate.

### Transcriptomic Analysis of Benzoate-Induced Genes Also Induced by Acid

The mechanisms that confer resistance to cytoplasmic acidification caused by permeant weak acids such as benzoate are likely to involve gene products that contribute to pH homeostasis. Respiratory neutralophiles, such as *B. subtilis* and *E. coli*, cope with cytoplasmic acidification and maintain pH homeostasis by catabolic consumption of acids that produce CO_2_ and alkaline amines [5]. Since growth in the presence of benzoate at pH 7 (Fig. 3B) and growth at external pH 6 (Fig. 2; Fig. 3D) are both conditions that depress cytoplasmic pH, it is expected that a high degree of gene expression overlap would occur under benzoate and acid stress. Earlier work with *E. coli* revealed protein expression correlations between an acid stress proteome [40] and other proteomes that exposed *E. coli* to permeant weak acids such as acetate [41] and benzoate [19]. A subsequent *E. coli* rapid acid shift microarray also identified genes important to acid resistance that respond to benzoate [42]. Comparison of our benzoate transcriptome with the steady-state *B. subtilis* acid transcriptome [31] yields gene overlap such that 42% of the genes significantly up-regulated by benzoate are also significantly up-regulated by acid. These genes are good candidates for response to and mediation of cytoplasmic acidification.

The acid stress genes up-regulated with benzoate adaptation include genes involved in catabolism, peptide transport, and drug resistance and transport (Table 1; Table 2). Most strinkingly, all of the formate dehydrogenases up-regulated at pH 6 (*fdhD, yjgCD, yrhEFG*) [31] were up-regulated by benzoate at pH 7. The formate dehydrogenases are capable of consuming acid coupled to proton export; they may play a role similar to that of the acid-induced H_2_ dehydrogenases in *E. coli* [43]. The acid-inducible glycine catabolism (*gcvPA*, *gcvPB*, *gcvT*) was also up-regulated by benzoate. Substrate decarboxylases are known to consume acid through CO_2_ removal [44]–[46]. Other enzymes, such as urease (UreABC) and GcvT of the glycine cleavage system, produce ammonia, which may assist in counteracting cytoplasmic acidification [47]–[49].

The low-pH-inducible *oppABCDF* [31] encodes an oligopeptide permease complex that imports short peptides including signaling peptides involved in sporulation and competence [50]. These peptides may also provide a source of amino acids for decarboxylases yielding basic amines that increase the cytoplasmic pH. Previous acid stress experiments in *E. coli* have identified similar amino acid transporters (*dppABCDF*) that are up-regulated at low pH [42], [51].

Drug resistance-related genes up-regulated either by benzoate at pH 7 or by low external pH include putative multidrug efflux transporters *yuxJ*, putative antibiotic/multidrug resistance proteins (*ywoG*, *yhcA*), MarR family transcriptional regulator homologs (*ywoH*, *yhbI*) as well as multidrug resistance loci *yhcA*, *yuxJ*, *ywoH*, and *ywoG* (Table 2). For comparison, in *E. coli* [52] and *Salmonella enterica* [53], expression of *marA*, a global activator associated with multiple antibiotic resistance, is induced by benzoate. Multidrug efflux pumps are known to respond to intracellular metabolites, pumping out toxic or undesirable metabolic products [54]. They could be used to export either the cytoplasmic benzoate, or at low external pH, the fermentation products such as acetate and formate driven back into the cell by the pH gradient. Multidrug transporters could also help maintain cytoplasmic pH; for comparison, in *E. coli* the MdfA multidrug transporter acts as a Na^+^(K^+^)/H^+^ antiporter at high pH [55].

Genes involved in sporulation, including *cotD*, a spore coat protein, and two genes required for entry into sporulation (*spo0E* and *spo0M*), were down-regulated by benzoate at pH 7, or by low external pH. These results are consistent with the observation that low external pH decreases sporulation efficiency [56]. Benzoate also up-regulated acid-inducible oxidative stress resistance genes including the Old Yellow Enzyme (OYE) family *yqjM* (*namA*) [57] and organic hydroperoxide resistance protein *ohrB* [58].

### Genes Induced Specifically by Benzoate

Some genes showed regulation specifically by benzoate, but not by low external pH. The genes most highly up-regulated by benzoate include the phenolic acid stress operon *yveFG*-*padC* operon. Benzoic and salicylic acids are catabolized by phenolic acid decarboxylase (*padC*) to vinyl phenol derivatives [22], [23]. The presence of benzoate also up-regulated *bsdC*, encoding another phenolic acid decarboxylase that has been suggested to aid in degradation and detoxification of phenolic acids and acts on benzoate derivatives such as vanillate and 4-hydroxybenzoate [10], [23], [59]. Since these enzymes are not acid-inducible [31], they more likely respond to benzoate anion accumulation. Benzoate also up-regulated acetyl-CoA synthetase, *acsA*, which is involved in acetate degradation [60]. Acetate catabolism could help protect the cell from cytoplasmic pH depression.

Urease genes (*ureA*, *ureC*) were induced specifically by benzoate, but not by low external pH. Ureases produce CO_2_ in the process of urea degradation and aid acid survival in some bacterial pathogens [61], [62]. For example, acid survival of the stomach-dwelling *Helicobacter pylori* depends on the CO_2_ and ammonia produced by its urease [63].

### Acid-Repressed Genes Down-Regulated by Benzoate

The most striking group of genes down-regulated by benzoate at pH 7 (and repressed at low external pH) was the alkali-shock regulon governed by SigW [27]. SigW-dependent genes are also repressed by sorbic acid [11]. We found that SigW governs eight of the genes most strongly down-regulated by benzoate (Table 1). The SigW regulon is considered to show “extracellular” response, mediated by proteolysis of the extracytoplasmic domain of anti-sigma RsiW [29], [30]. Nevertheless, the mechanism also involves a cytoplasmic interaction between SigW and RsiW, also involving the transmembrane protein YluC. Thus it is possible that SigW actually detects cytoplasmic pH elevation, as the consequence of alkali shock, rather than external high pH directly. The benzoate repression of SigW confirms our interpretation that the overlap between benzoate and external-pH transcriptomes represents a cytoplasmic pH transcriptome.

Cell wall-associated proteins *wapA* and *wprA* were also significantly down-regulated by benzoate. WprA is a cell wall protein precursor that is cleaved and processed to form two cell wall-bound proteins, one of which is known to be a protease and could be involved in the necessary cell wall elongation that occurs during growth [64].

### Genes Regulated by External pH but Not Benzoate

Acid-inducible genes showing no significant response to benzoate included oxalate decarboxylase, *oxdC* [65], [66]; acetoin production, *alsDS*; several substrate dehydrogenases (*maeA*, *gabD*, *ydaD*, *ytbE*, *yvfV*); and polyamine biosynthesis, *speA*, *speBE* [31]. We propose that these systems have regulators that sense low pH outside the cell membrane, not in the cytoplasm. For comparison, the well-studied amino acid decarboxylase systems in *E. coli* such as lysine decarboxylase *cadA* are regulated by a membrane protein (CadC) whose periplasmic domain senses pH [67].

In terms of stress regulons, the acid-inducible SigX regulon [31] did not respond to benzoate. Benzoate did not repress the alkali-inducible SigH and SigL regulons.

### Cluster Analysis

Our cluster analysis of the benzoate adaptation and low-pH adaptation transcriptomes confirms the finding of substantial overlap between benzoate and low-pH response (Fig. 5; individual genes shown in Table S1C). Within the largest cluster (up-regulated by benzoate and at low pH) we identified two subsets. The larger subset consists of genes up-regulated more by benzoate than at low pH. This group includes the oligopeptide transport system (*oppABCDF*), all of the formate dehydrogenases identified (*fdhD, yjgCD, yrhEFG*), the glycine decarboxylase system (*gcvPA-gcvPB-gcvT*), the TCA cycle (*citZ-icd-mdh*, *sucCD*), and branched-chain amino acid synthesis (*ilvBHC-leuAB*) (Table S1C). The smaller subset consists of genes up-regulated more at low pH than with benzoate. This group includes the potassium/cation transport system (*czcDO*), an oxidative stress protein (*ohrB*), and a catalase (*katX*). The greater magnitude of most of the benzoate expression ratios suggests that, under our experimental conditions, benzoate adaptation at pH 7 caused greater cytoplasmic pH stress than did growth at pH 6.

### Comparison with Salicylate and Sorbate Transcriptomes

Our benzoate transcriptome was compared with the reported transcriptome of salicylic acid addition (Table S1A) based on data reported in Ref. [10]. Comparison was limited by the many differences in experimental design, most notably the salicylic acid time course which induced general stress responses (CtsR, SigB); the concomitant external-acid shock; and the use of a different growth medium. The main benzoate-up-regulated genes that also appear after salicylate-acid-shock include those proposed for salicylate detoxification (*yhcC*, *yuiA*, *ynfC*, *yfmP*), urease (*ureA*, *ureC*) and cell wall-associated *wapA*. The induction of the SigB general stress response in the salicylate transcriptome may have more to do with the rapid drop in external pH from pH 7 to pH 5.5 when salicylate was added [10]. Previous work determined that the SigB general stress response is activated upon a rapid acid shift from pH 7.5 to pH 6.0, with transcription of *sigB* reaching a peak at 15 min after acid shock and dropping off significantly at 20 min [68], and more moderately activated in steady-state low pH conditions (pH 6 compared to pH 7) [31], but the response is not activated by sorbic acid stress at pH 6.4 [11] or by the addition of 5 mM HCl at pH 5.5 [10]. Our transcriptome displayed minimal expression of genes in the SigB regulon (20 out of 127 genes identified [69]), many of which can be regulated by other mechanisms. The observation of benzoate adaptation rather than benzoate shock focuses on the more persistent components of adaptation to a permeant weak acid.

The effect of benzoic acid on the cell wall and membrane composition was similar to but not as pronounced as that of sorbic acid [11], another lipophilic permeant weak acid. Seven BkdR-dependent genes of the *bkd* operon (*bkdB*, *bkdBB*, *bkdBA*, *bcd*, *buk*, *lpdV*, and *ptb*), which are involved in the synthesis of branched-chain fatty acid precursors, were significantly up-regulated, indicating a possible remodeling of the cell membrane to include more branched-chain fatty acids [11], [70].

Our study reveals the details of the cytoplasmic pH response to external acidification and to benzoate stress at neutral pH. *B. subtilis* showed remarkably robust adaptation to benzoate at high concentrations. We present a benzoate transcriptome and use it to distinguish between external versus cytoplasmic pH dependence of pH stress genes. We highlight important acid reversal mechanisms, such as formate dehydrogenases and amino acid transporters, that appear to respond to cytoplasmic acidification; as well as other mechanisms, such as amino acid decarboxylases that respond to external acid but not benzoate. And the unexpected repression of the SigW regulon by benzoate adds important information for elucidating the mechanism of this alkali-shock response.

## Materials and Methods

### Strains and Plasmids

The GFPmut3b plasmid pMMB1311 was constructed from pBSVG101, which encodes a version of GFP expressed from the bleomycin resistance promoter (P*_bsr_*). Plasmid pBSVG101 was purified from *B. subtilis* strain BEST215 [71], transformed into Dam^+^ *E. coli*, and re-purified. The GFP allele was changed to GFPmut3b [34] by QuikChange site-directed mutagenesis using PfuUltra II Fusion HS DNA Polymerase (Stratagene) and primers based on the GFPmut3b sequence. The reaction was transformed into *E. coli* strain AG1111 (MC1061 F'*lacI^q^ lacZ*M15 Tn*10*). Plasmid from a brightly fluorescing transformant (MMB1309) was sequence-verified to confirm the GFPmut3b mutation. This plasmid, pMMB1309, was then introduced into the *B. subtilis* strain AG174 (JH642, *trpC2 pheA1*) by natural transformation [72] to create strain MMB1311. In strain MMB1311, pMMB1309 allows for constitutive expression of GFPmut3b from the P*_bsr_* promoter and contains both *B. subtilis* and *E. coli* origins of replication.

### Fluorimetry Measurement of Cytoplasmic pH

GFPmut3b fluorimetry of whole-cell suspensions was based on the method of Wilks and Slonczewski (2007) [35], using pH-dependent excitation spectra [73], [74]. *B. subtilis* MMB1311 was cultured overnight for 16 to18 hr rotating at 37°C in unbuffered LBK medium (Luria broth with 100 mM KCl replacing NaCl) [51]. In order to maintain the plasmid, overnight cultures included 5 µg/ml tetracycline. Overnight cultures were diluted into buffered LBK medium in pre-warmed 250-ml baffled flasks; the dilutions, buffers, and pH depended on the experiment. Bacteria were cultured at 37°C rotating at 240 rpm to an optical density at 600 nm (OD_600_) of 0.2 to 0.3. To diminish background fluorescence, cultures were resuspended at OD_600_ 0.4 in buffered M63 salts medium supplemented with casein hydrolysate (7.45 g/liter KCl, 2 g/liter casein hydrolysate, 2 g/liter (NH_4_)_2_SO_4_, 0.4 g/liter KH_2_PO_4_, and 0.4 g/liter K_2_HPO_4_) [75]. The use of M63 medium avoids fluorescent contaminants present in LBK.

For fluorimetry, excitation spectra were recorded using a Fluoromax-3 spectrofluorimeter (Horiba Jobin Yvon) as described previously [35]. Spectra were recorded for three biological replicates for each condition. For time course experiments, continuous excitation spectra were recorded at 4-s intervals. Emission was set at 545 nm (slit width, 20 nm), and excitation was recorded at 480 nm to 510 nm (slit width, 2 nm) [34]. Fluorescence intensity data were converted to cytoplasmic pH by interpolation between two pH measurements following the addition of 10 µM nigericin, a H^+^/K^+^ ionophore that collapses ΔpH across the membrane [38]. All cultures for fluorimetry were maintained at 30°C for stability of the GFPmut3 protein.

In order to establish the relationship between pH and fluorescence intensity in living *B. subtilis* cells (Fig. 1), strain MMB1311 was cultured in buffered LBK (50 mM MOPS, pH 7.5) to OD_600_ 0.2 and resuspended at OD_600_ 0.4 in supplemented M63 medium with 10 µM nigericin. Buffers included in growth and resuspension media were as follows: 10 mM Homopiperazine-N,N′-bis-2-(ethanesulfonic acid) (HOMOPIPES), pH 5.7; 10 mM MES, pH 6.1; 10 mM piperazine-N,N′-bis(ethanesulfonic acid) (PIPES), pH 6.6; 10 mM MOPS, pH 7.0; 10 mM MOPS, pH 7.5; and 10 mM N-Tris(hydroxymethyl)methyl-3-aminopropanesulfonic acid (TAPS), pH 8.0. Fluorescence intensity was summed over the excitation range.

### cDNA Preparation and Array Hybridization

*B. subtilis* AG174 (obtained from Alan Grossman) was cultured overnight at 37°C in unbuffered LBK. Bacteria were diluted 500-fold into pre-warmed 250-ml baffled flasks containing 15 ml of LBK buffered with 50 mM MOPS, adjusted to pH 7.0 using KOH. Sodium benzoate was added to the media prior to inoculation at a concentration of 30 mM. For each experimental condition (presence or absence of 30 mM benzoate), four independent cultures were rotated at 240 rpm at 37°C. Cultures were harvested at early log phase (OD_600_ 0.2). Samples were transferred from growth culture to sterile vials containing 1 ml ice-cold 10% phenol-ethanol stop solution [76] in order to stabilize the bacterial RNA. RNA was isolated as previously described [31], [42], [43], [51] using the RNeasy Kit with on-column DNA digestion (Qiagen). The RNA isolation protocol for gram-positive microorganisms was followed, in which 3 mg/ml of lysozyme is used to degrade the bacterial cell wall. Isolated RNA concentration and quality was examined for each sample using the Agilent Bioanalyzer 2100.

Standard methods were used for cDNA synthesis, fragmentation, and end-terminus biotin labeling [31], [43], [51]. Labeled cDNA samples were hybridized to Affymetrix GeneChip *B. subtilis* antisense genome arrays [77]. Hybridized arrays were stained with streptavidin-phycoerythrin using the Affymetrix Fluidic Station 450 and then scanned with a GC3000 scanner. The GeneChip *B. subtilis* arrays include 4,351 probe sets that cover 4,095 protein-encoding open reading frames (ORFs) (approximately total complete genomic coverage) as well as 606 probe sets covering intergenic regions annotated to the RefSeq genome sequence, NC_000964 (further details in Table S1A).

### Analysis of Gene Expression

Benzoate-dependent expression ratios were determined by a model-based expression analysis of the probe-level data from Affymetrix's CEL files. Analysis was performed using dChip software (http://www.dchip.org) as described previously [31], [43], [51]. In the dChip model, a linear function relates target RNA levels to the probe signals by weighting the significance of all probes for each gene. Each array was normalized to an array of median brightness, using local regression on an invariant set of probes [78]. Only the perfect match probes were used to calculate model-based expression indices.

For each condition, the dataset included four biological replicates (independent with respect to *B. subtilis* growth, RNA isolation, sample preparation and array hybridization). Statistical Analysis Software (SAS) was used to test for significant differences in expression between presence or absence of benzoate. A two-sample t-test was performed on the log_2_-transformed model-based expression indices on a gene-by-gene basis at a significance level of 0.001, thus expecting a 1 in 1000 false positive rate [31], [43], [51]. For a gene showing average within-group variability, our sample size (four replicates with 30 mM benzoate and four replicates without benzoate, 4,351 ORFs) provided statistical power of 98% to detect a 2-fold difference in gene expression between experimental conditions.

All probes that showed significant benzoate effects on expression (including duplicate probes for some ORFs) are included in Table S1A. The intergenic regions showing benzoate-dependent expression are tabulated in Table S1B.

Normalized expression ratios of the benzoate transcriptome and the earlier acid array [31] were analyzed using unsupervised, average-linkage hierarchical clustering with uncentered Pearson correlation as a similarity metric, performed with Cluster 3.0 [79]. Only the 280 probe sets with significant expression ratios were chosen to be visualized with Java TreeView [80]. The cluster analysis output list and a large, gene-annotated heat map are available in Table S1C.

### Real-Time Quantitative Reverse Transcription-PCR

Expression of mRNA for individual genes was quantified by real-time PCR using an ABI Prism7500 DNA analyzer (Applied Biosystems) as described previously [42]. For each growth condition, three independent biological replicates were tested. Primers used are presented in Table S1D. The SYBR Green PCR one-step reverse transcription-PCR protocol (Applied Biosystems) was used, in which cDNA reverse transcription and PCR amplification occur in the same well. Nucleic acid concentrations were as follows: 0.1 nM forward primer, 0.1 nM reverse primer, and 50 ng target RNA. PCR cycling conditions were as follows: reverse transcription at 48°C for 30 min and 95°C for 10 min, 40 cycles of denaturation at 92°C for 15 s, and extension at 60°C for 1 min. The total RNA in each sample amplified was used as the basis to normalize individual gene expression profiles. All expression levels are presented relative to the expression in the 0 mM benzoate controls.

## Supporting Information

Table S1 A) Expression indices and expression ratios showing significant benzoate regulation. B) Intergenic regions significantly regulated by the presence of benzoate. C) Result of cluster analysis. D) Real-time PCR primer sequences.
(0.22 MB XLS)
